# Psilocybin-Assisted Compassion Focused Therapy for Depression

**DOI:** 10.3389/fpsyg.2022.812930

**Published:** 2022-03-25

**Authors:** Wendy Pots, Farid Chakhssi

**Affiliations:** ^1^Arjuna Labs, Mill Valley, CA, United States; ^2^Bureau Apeneus, Zutphen, Netherlands

**Keywords:** psilocybin, psychotherapy, treatment protocol, compassion focused therapy (CFT), depression, psilocybin-assisted psychotherapy

## Abstract

Psilocybin-assisted psychotherapy, i.e., psilocybin treatment with psychological support, has demonstrated the efficacy of psilocybin to reduce depressive symptoms. However, in clinical trials, the structure of psilocybin-assisted psychotherapy is primarily based on preparation, navigation (support during dosing sessions), and integration. For psychotherapeutic guidance, the application of this structure is favored over the usage of theoretical models. The applied psychotherapeutic models may be of critical importance if the effects are augmented due to the psychologically insightful experiences during the navigation and integration sessions. One of the important next steps is to provide therapists with guidance on how to provide psilocybin-assisted psychotherapy. We present an integrated protocol for psilocybin-assisted psychotherapy for depression based on the theoretical model and psychotherapeutic framework of Compassion Focused Therapy (CFT). We hypothesize that CFT can provide the theoretical model and compassion practices that will reinforce the experiences during the navigation and follow-up therapy sessions. In this paper, we describe the rationale for selecting CFT, the compatibility of CFT and psilocybin-therapy, an overview of the psilocybin-assisted CFT protocol, the study protocol, and limitations to this approach.

## Introduction

Major depression is a prevalent disorder that has a large impact on quality of life and is associated with high rates of comorbidity and increased mortality (Vigo et al., 2016). Current treatment approaches for depression, including psychotherapies and pharmacotherapies, are effective but leave significant room for improvement since remission is only achieved for less than half of the treated patients (Cuijpers et al., 2020).

More recently, psilocybin has gained attention as a new paradigm in the treatment of depression. Psilocybin, a classic hallucinogen, effects glutamate levels in key areas of the brain, including the medial prefrontal cortex and hippocampus that are associated with positive changes in self-experience and increased feelings of unity with others and one’s surroundings (Nour and Carhart-Harris, 2017; Mason et al., 2020). Distortions of self-experience may be an important mechanism to target in the treatment of depression (Carhart-Harris and Goodwin, 2017).

Several studies have demonstrated the efficacy of psilocybin with moderate to large effect sizes in patients with life-threatening cancer and depressive symptoms (Griffiths et al., 2016; Ross et al., 2016), and in patients with major depressive disorder (Carhart-Harris et al., 2016; Davis et al., 2021). These findings also show promising tolerability data, eliciting head-to-head comparative efficacy studies with current treatments (e.g., current pharmacotherapies). Furthermore, psilocybin has a lower risk for addiction and harmful neurological effects compared to other novel interventions such as ketamine (Morgan et al., 2012; Fond et al., 2014; Johnson et al., 2018; Mcintyre et al., 2020).

Psilocybin treatment with psychological support, structured in preparation-, navigation- and integration sessions, has shown to act rapidly and to have long-lasting effects on depression (Carhart-Harris et al., 2016; Agin-Liebes and Davis, 2021; Davis et al., 2021). A recent review of psychotherapeutic components in psilocybin-assisted psychotherapy found that psychotherapy was primarily given in the preparation and integration sessions, and that psychotherapy mostly consisted of a non-directive approach and music therapy (Horton et al., 2021). The core elements of supportive psychotherapy are calmness, empathy, personal support, and reassurance (Johnson et al., 2008). Some studies have combined psilocybin with Cognitive Behavioral Treatment (CBT) for smoking cessation (Johnson et al., 2017), Acceptance and Commitment Therapy (ACT) for treatment-resistant depression (Sloshower et al., 2020; Watts and Luoma, 2020), or Motivational Enhancement Therapy (MET) for alcohol dependence (Bogenschutz et al., 2015). In most studies, the therapeutic content is often neither reported nor examined. Therefore, it remains unclear how psychotherapy affects the patient’s experience. However, it has been suggested that the offered psychotherapeutic model enhances the personal and meaningful experiences, and thereby the antidepressant effects, of psilocybin-assisted treatment (Griffiths et al., 2006, 2011). One of the important next steps is to provide therapists with guidance on how to provide psilocybin-assisted psychotherapy (Nutt and Carhart-Harris, 2021), including the development of treatment protocols (Sloshower, 2018; Gorman et al., 2021), and support that with research into the possible added value of psychotherapy or psychological support to the effect of psilocybin for depression.

A recently developed therapeutic approach that is of particular interest for psilocybin-assisted therapy is Compassion-Focused Therapy (CFT; Gilbert, 2014). CFT provides patients with a rationale and practices that might enhance the effects of psilocybin-assisted therapy by increasing connectedness, compassion for self, and compassion for others. CFT is based on evolutionary psychology and attachment theory and stimulates the affiliative or “rest and digest” system (Gilbert, 2014). It enables insight into the interplay of the lack of care and compassion, especially early in life, and underlying mental health problems, and shows how developing compassion can act as a psychotherapeutic process and promote social connection and social safeness (Weng et al., 2013; Gilbert, 2020). Previous studies suggest that certain substances, including psilocybin, can produce similar increases in self-compassion and reductions in self-criticism as compassionate practices (Kamboj et al., 2015; Watts et al., 2017). Likewise, research shows that psilocybin-assisted psychotherapy may decrease negative affect and the neural correlates of negative affect (Davis et al., 2021) and increase connection and acceptance (Watts et al., 2017), similar to the proposed working of the affiliative system within CFT. We believe that CFT may be a relevant psychotherapeutic model to help to improve the understanding of the experiences during administration or navigation and integration sessions, while at the same time providing meaningful practices that help the integrative process.

Therefore, the aim of the current article is to provide a CFT framework in which psilocybin-assisted psychotherapy can be embedded.

## Compassion Focused Therapy

Compassion focused therapy is specifically developed for and aimed at individuals who have a compromised capacity for experiencing and expressing affiliative motives and emotion to self and others (Gilbert, 2010). It is particularly suited for individuals characterized by the tendency to negatively evaluate and judge aspects of the self, such as patients with depression. Several meta-analyses demonstrate efficacy for compassion-based interventions in various (non)-clinical populations with moderate to large effects on depression and well-being (Leaviss and Uttley, 2015; Kirby et al., 2017; Wakelin et al., 2021).

**FIGURE 1:**
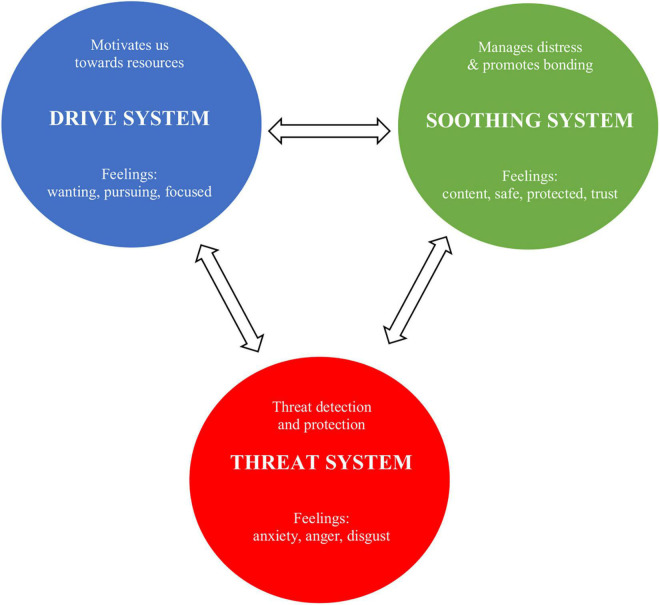
Three types of CFT emotion regulation systems.

Compassion focused therapy was developed as a transdiagnostic model by Paul Gilbert (Gilbert, 2005) in response to the observation that many of his chronic depressed clients, in particular those high in shame and self-criticism, did not benefit from traditional therapy. Although these clients were able to engage in cognitive and behavioral exercises, they weren’t able to generate a self-compassionate inner voice. Gilbert (2014) proposed a framework for the biological mechanisms underpinning compassion that is based on evolutionary biology. CFT takes an evolutionary functional view to emotion—especially the affiliative emotions and their competencies—and focuses on three major emotion regulation systems: (1) the threat protection system which responds to threat with defense strategies; (2) the drive- and resource-seeking system which links to achievement and acquiring of resources and rewards; and (3) the soothing and affiliation system which enables individuals to soothe, calm, and content themselves (Gilbert, 2014). The three major emotion regulation systems are displayed in Figure 1. Compassion is thus understood as an evolved motivational system designed to regulate negative emotions through attuning to the feelings of self and others, and expressing and communicating feelings of affiliation, warmth, and safeness (e.g., Gilbert, 1989; Spikins et al., 2010). Within the framework of treating depression, harmonizing the emotion regulation system is essential as studies show that emotion regulation affects depressive symptoms *via* increasing rumination and decreasing reappraisal (Joormann and Stanton, 2016).

Gilbert’s (1989) evolutionary model suggests that the potential for compassion evolved with the affiliative system that is linked to the attachment system. CFT is underpinned by social mentality theory (SMT), which states that different mental states not only organize our minds but also shape our relationships (Gilbert, 1989, 2000, 2005). Both care seeking and care giving social mentalities are activated when one is relating to others (e.g., a crying child and a comforting mother), but can also be activated when relating to the self. Compassion, as a social mentality, can “flow” in three directions. First, there is the compassion we can feel for others; second, there is the compassion we can feel coming from others to ourselves, and thirdly there is the compassion we can direct to ourselves which is defined as self-compassion. Each of these can be a focus in CFT. Research shows that the quality of care and affection received in childhood lays the foundation for being caring and compassionate as an adult (Gillath et al., 2005). Imagining being cared for and receiving compassion has the same effects, as it stimulates the soothing system, helping individuals feel safe and calm. CFT emphasizes the significance of our affiliative system in reducing the activity in the threat-based protection system by allowing us to feel cared for and to be able to offer care to both ourselves and others (Gilbert, 2014). Accordingly, CFT targets both the psychological and the biological processes underpinning care and compassion. When combined these processes help to down-regulate the threat system and stimulate positive affect and a sense of social safeness.

The primary aim of CFT is to help clients develop these caring processes and create a better sense of social connectedness to understand and respond to their distress from the perspective of a compassionate mind. Through the incorporation of a range of compassion-based skills, attributes, and qualities, clients become more distress aware and insightful, learning how to develop empathy and distress tolerance and how to take wise and compassionate action to address distress. Thus, in this model, the skill of giving or receiving compassion can be enhanced through therapy and generate experiences of being cared for and socially connected to (a) caring other(s).

## Rationale for Compassion Focused Therapy as a Therapeutic Model in Psilocybin-Assisted Psychotherapy

Conventional Cognitive Behavioral Therapy (CBT) approaches to depression primarily target signs and symptoms of depression, but do not impact emotional, psychological and spiritual wellbeing as much as more recent approaches such as CFT (Van Agteren et al., 2021). CFT focuses more on the persons’ relationship to thought and emotion rather than on their content, and uses techniques and concepts derived from mindfulness, emotion focused therapy, psychodynamic therapy, and attachment theory (Gilbert, 2020). More specifically, CFT targets compassion as a psychotherapeutic process and effectively increases social connection and social safeness (Leaviss and Uttley, 2015; Kirby et al., 2017; Craig et al., 2020). Weng et al. (2013) showed that compassion can be cultivated through training compassion meditation and imagery, boosting the brain’s resilience to suffering. This is important because part of the reason that compassion can be helpful is that it is activating particular physiological systems linked to caring, social connections, and feeling cared for Gilbert (2020), Holze et al. (2021), and Steffen et al. (2021).

Similar to CFT, psilocybin-assisted psychotherapy is aimed to increase openness and acceptance by creating space for people to tolerate and allow internal and external discomfort and enhancing compassion for self and others. Indeed, a study by Barrett et al. (2020) showed that psilocybin may decrease negative affect and the neural correlates of negative affect. One-month post-psilocybin administration, positive affect remained elevated and trait anxiety was reduced (Barrett et al., 2020). They hypothesized that the reduction of negative affect may undermine ruminative processes that contribute to the development and maintenance of mood disorders. Further studies provide evidence that psilocybin has a significant effect on social connection, attachment, and feeling more connected to self, others, and nature/the world (Watts et al., 2017; Forstmann et al., 2020), similar to the proposed working of the soothing system within CFT (as described above). Furthermore, several authors (e.g., Watts et al., 2017) have recommended that social connection as flows (from self, others, and the world), should be examined in future studies as they appear to form a central theme of patient’s experiences. This also adds further rationale to the use of CFT as it facilitates the flow of compassion.

As people with depression tend to operate from their threat system by having a negative affect and thoughts, providing psilocybin within the framework of CFT will stimulate the affiliative system and provide a positive and supposedly healing context of thoughts, feelings, and behaviors.

## Psilocybin-Assisted Compassion Focused Therapy Protocol

The current therapy protocol was constructed based on the basic structure of other psychedelic-assisted psychotherapy protocols (Johnson et al., 2014; Mithoefer, 2015; Guss et al., 2020; Watts, 2021). The included interventions and exercises were devised in conjunction with the CFT model. The psilocybin-assisted CFT protocol consists of 13 sessions (see Table 1). In the protocol, each session includes instructions for the therapist to guide the treatment session. Also, each session includes psychoeducation (e.g., the conceptualization of depression) followed by an in-session experiential exercise (e.g., soothing rhythm breathing; see below for an explanation), which participants are also asked to practice on their own between sessions (2 h per week). Compliance with the homework sessions is assessed at the start of each session.

**TABLE 1:** Overview of CFT-based psilocybin-assisted psychotherapy sessions in treatment protocol.

Session information	Week	Session number	Duration hours (h)	Session goals and CFT components
Preparatory session	1	1	1.5	Establishing a therapeutic alliance. Psychoeducation CFT model and psilocybin experience. Practicing mindful breathing. Compassionate motivation.
Preparatory session	2	2	1.5	Tricky brain and Emotion regulation systems. Practicing compassion focused body practices. Practicing compassionate flow. Case conceptualization.
Preparatory session	3	3	1.5	Compassionate self. Multiple selves. Intention setting medication session 1.
Psilocybin navigation session	4	4	7–8	Therapists gather information for CFT based case conceptualization. Therapeutic stance is largely non-directive, allowing the individual’s own healing intelligence determine what will happen and following the participants lead. Working with parts is appropriate when participants spontaneously bring them up.
Integration session	4, 1 day after session 4	5	1.5	Debriefing of medication session and reconstruction of narrative of experience. Identify and explore association of core experiences with CFT principles. Practicing compassionate skills and compassionate reframing.
Integration session	5	6	1.5	Deepening of compassionate self and compassionate skills. Practicing relating compassionate self to multiple selves and applying compassionate skills in daily life.
Integration session	6	7	1.5	Deepening of compassionate self and compassionate skills. Practicing relating compassionate self to multiple selves and applying compassionate skills in daily life.
Preparatory session	7	8	1.5	Adapting case conceptualization with recent information from previous sessions. Practice compassionate skills. Intention setting medication session 2.
Psilocybin navigation session	7	9	7–8	Therapists gather information for CFT based case conceptualization. Therapeutic stance is largely non-directive, allowing the individual’s own healing intelligence determine what will happen and following the participants lead. Working with parts is appropriate when participants spontaneously bring them up.
Integration session	7, 1 day after session 9	10	1.5	Debriefing of medication session and reconstruction of narrative of experience. Identify and explore association of core experiences with CFT principles. Recap self-compassion in the body. Compassionate letter writing.
Integration session	8	11	1.5	Deepening of compassionate self and compassionate skills. Practicing relating compassionate self to multiple selves and applying compassionate skills in daily life. Practice problem solving and daily living with a compassionate mind.
Integration session	9	12	1.5	Deepening of compassionate self and compassionate skills. Practicing relating compassionate self to multiple selves and applying compassionate skills in daily life. Practice values-based living.
Follow-up session	10	13	1.5	Exploration of insights gained from medication sessions and integration sessions. Reinforce CFT concepts and encourage corrective behavioral changes. Review CFT skills and create a progress and contingency plan.

### Compassion Focused Therapy-Based Preparatory Sessions

**FIGURE 2:**
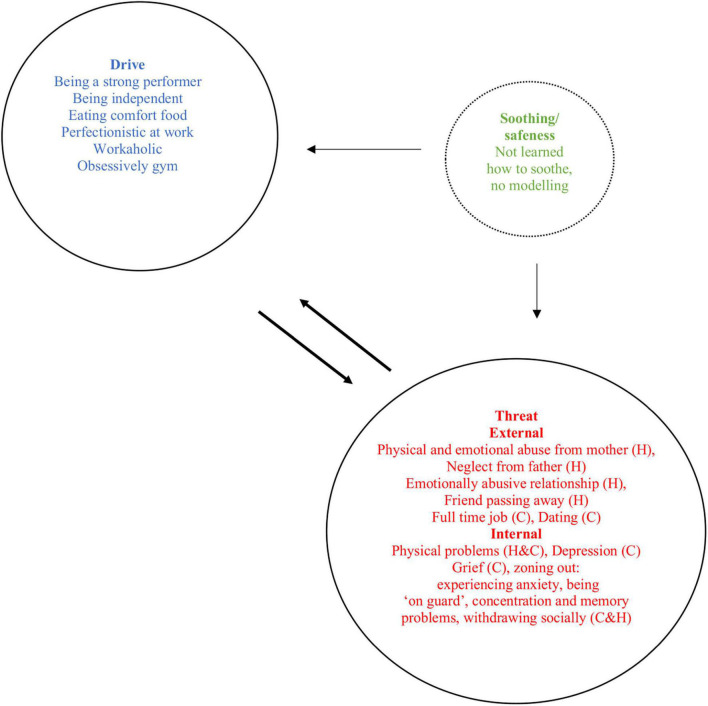
Example of CFT emotion regulation systems case conceptualization [based on Welford (2010)]. H, history; C, current.

Within the psilocybin-assisted CFT protocol, CFT is the framework to provide the understanding of the nature of depression and its thoughts, feelings, and behaviors, as well as the psychedelic experiences. The goal of the preparatory sessions is for the therapists to help participants understand and respond to their distress from the perspective of a compassionate mind, generating an understanding that comes from an evolutionary perspective of the mind. Therefore, in the preparatory sessions (1–3), therapists will frame the conceptualization of depression based on the affiliative motives and emotions of the participant, and explain the concept of the so called “Tricky Brain.” This tricky brain encompasses the notion that all human beings find themselves with an evolved brain and influential environments that we didn’t choose but shaped our responses and behaviors. Within CFT, the concept of the tricky brain is used as a way to de-shame participants: “*it is not our fault that we have this highly evolved brain that can create these fear states, nor is it our fault that we are born in these particular circumstances that gave us these learning behaviors*.” This concept of the tricky brain will be explored, as well as the fear of compassion. Therapists will explain the evolved functions of emotions by using the three circles model: the threat system, the drive system, and the soothing system, drawing them with examples from the life of the participant (see Figure 2 for a case example).

Soothing rhythm breathing (i.e., a breathing technique with a style and rhythm of breathing that feels calming and soothing) and compassion focused body practices (i.e., exercises aimed at improving and utilizing body awareness to support a compassionate mindset) are taught in the sessions together with the therapist and are given as homework. During the first three preparatory sessions, the therapist pays close attention to the way the participant perceives him or herself, being attentive to the different emotional states that may lie underneath different affiliative motives and emotions, and to thoughts and feelings about the psychedelic experience. Exercises focusing on building a compassionate self and working with multiple “selves” or “parts” will be explored to give context to the daily life struggles of the participant. Within CFT, working with those parts is a therapeutic technique that helps people access and relate to the different emotional states they experience in their daily life from the perspective of the compassionate self. For example, while tuning into the thoughts, bodily feelings, action tendencies, memories, and needs of a particular part (i.e., anxious, sad, or angry) that comes up, the therapist can ask: “*From the wisdom of the compassionate self, what is your view of that* [anxious, sad, angry, …] *part of you? What do you think it wants, and what do you think it fears? How would you like to relate to that part of you?*” The compassionate self can relate to that particular self that is explored from the qualities of wisdom, strength, and commitment. At the end of the third preparatory session, the result of these explorations will be translated into an intention for the first psilocybin navigation session. The preparation for the second psilocybin navigation session will integrate the explorations with the insights and/or learnings from the first psilocybin navigation session.

### Compassion Focused Therapy-Based Navigation Sessions

The psilocybin navigation sessions are guided by a non-directive approach. The non-directive approach has been a core principle of psychedelic-assisted therapies as it enables a patient to adopt an uncritical approach concerning their own psychedelic experience(s) and engage in self-directed insight-finding and meaning-making processes (Gorman et al., 2021). It allows the client to reconcile unconscious material in the order and nature in which it comes to the surface for healing. The navigation sessions will be guided with music generated by the therapist or curated psychedelic therapy playlists that are freely available (e.g., by Mendel Kaelen^1^). Within the context of inner healing during the navigation session, one or multiple parts can come up. When the participant is being burdened or hindered by that particular self, the therapist will encourage the participant to look into their own inner experience for insights and guide him or her to go into the experience with their inner healing intelligence (Mithoefer, 2015; Clare, 2018; Phelps, 2019). The experience of the participant can be further guided from the understanding of CFT and working with different parts. The participant can be guided toward bringing in the compassionate self and relating to the different parts from the position of the compassionate self as explained above. The CFT case conceptualization is consistently held as a temporary framework of understanding and used iteratively as it occurs throughout all therapeutic encounters. Thus, the case conceptualization will continuously be updated by information from the navigation sessions.

### Compassion Focused Therapy-Based Integration Sessions

Integration sessions are partly debriefing of the psychedelic experiences of the psilocybin navigation session, and partly psychotherapeutic CFT integration sessions. The debriefing focuses on recollecting the experiences of the psilocybin navigation session and the accompanying feelings. All psychotherapeutic sessions focus on deepening the compassionate self and applying compassionate skills in daily life. Participants are taught how to engage with themselves and others with compassion, teaching them the skills to develop the key aspects of compassion. For example, practices of compassionate attention help to refocus attention to positive attributes or skills, such as courage, kindness, or gratitude; practices of compassionate behavior focus on practicing new behavior with an encouraging, warm tone in their mind; and compassionate imagery practices focus on exploring the image of a compassionate self, including facial expressions, body postures, and voice tones. Exercises are done in sessions and given as homework: compassionate thought challenging records, as a way to reframe thoughts and feelings from the compassionate mind; compassionate letter writing, where participants will write about painful events from a compassionate reframing; worksheets that address the inner critic.

### Compassion Focused Therapy Therapeutic Stance

Although all psychotherapy sessions are didactic, all sessions are equally experiential as they contain exercises of CFT. The therapeutic stance of CFT is to offer a safe therapeutic setting that will allow the participant to freely and safely explore the psychedelic realm and the therapeutic relationship, to tolerate the distress that accompanies a participant’s journey and therapy, and to be non-judgmental and approach inner-criticism with kindness.

## Study Protocol and Therapist Training

Overall, research indicates that psilocybin-assisted psychotherapy shows promising results regarding the acceptability and effectiveness for patients with depression. However, it is currently not clear what psychotherapy or even standard psychological support adds to the effect of psilocybin for depression. This question could be addressed in a three-armed randomized clinical trial (RCT), comparing psilocybin-assisted CFT to psilocybin-assisted psychotherapy with non-directive psychological support (see Griffiths et al., 2016) and psilocybin-assisted psychotherapy with minimal support (psilocybin-only condition). In the psilocybin-only condition, because of potential adverse effects such as crying, sadness, or grief (Davis et al., 2021), some additional safety measures should be implemented. Before and during psilocybin administration trained research assistants guide participants and intervene when participants are in distress by providing verbal or physical reassurance (see Griffiths et al., 2016). We hypothesize that both the psilocybin-CFT condition and the psilocybin-standard condition would have more favorable changes, compared to the psilocybin-only control condition, and that psilocybin-assisted CFT would result in a larger effect than psilocybin-assisted psychotherapy with non-directive psychological support.

An alternative to a three-armed RCT for settings where group designs are challenging or costly to perform would be a single case experimental design (SCED). SCEDs are designs that do not require large groups while permitting to draw scientifically valid inferences about the effects of an intervention and their theoretical mechanisms (Kazdin, 2019). In particular, a single case multiple baseline design (which is a variant of a SCED) would be a feasible and appropriate design to measure the efficacy of the psilocybin-assisted CFT protocol for depression. In a multiple baseline design, data is repeatedly collected during a baseline phase on outcomes of interest (e.g., well-being or depression) to describe the level of functioning. After assuring that the functioning is stable in the baseline phase, the intervention will be introduced to the participant. Data collection is continued during the intervention, and if the intervention is effective, changes are expected in the outcomes of interest. This suggests that the intervention was responsible for the changes in the outcomes. By varying the length of the baseline for the participants (i.e., one participant will start after 2 weeks, whereas another participant after 3 weeks of gathering baseline data), the influence of other factors (such as time effects) is unlikely. The repeated change in outcomes in response to the introduction of the intervention demonstrates its efficacy (Kazdin, 2019). In other words, if changes in the participant’s functioning occurred during or after the psilocybin-assisted CFT intervention, these changes are seen as evidence of the effectiveness of the specific intervention.

The proposed primary outcome measure is the Hamilton Depression Rating Scale (GRID-HAMD), in line with Davis et al. (2021). Secondary outcome measures could be the Mental Health Continuum-Short Form (MHC-SF; focuses on emotional, psychological, and social well-being), Compassion Engagement and Action Scales (CEAS), the Types of Positive Affect Scale (TPAS; subscale Safe Positive Affect), and the Fears of Compassion Scales (FCS). Physiological assessment is measured with HRV assessment of resting state and activity state (Steffen et al., 2017; Caldwell and Steffen, 2018). All outcomes could be administered at baseline, before psilocybin sessions at week 4, and week 7, and at a follow-up session at week 10.

To examine the psilocybin-assisted CFT protocol in a study, we are devising a treatment manual and training for therapists based on recent CFT handbooks and/or manuals (Arnold et al., 2021; Cattani et al., 2022; Gilbert and Simos, 2022). The goal of the training is to introduce therapists to the core principles of psychedelics and CFT and to train them how to use the therapist manual.

## Discussion

In this paper, we provided a CFT framework for psilocybin-assisted psychotherapy for depression. We believe that such a framework is needed as it may enhance the efficacy of psilocybin-assisted psychotherapy for depression. Offering a psychedelic experience within the framework of CFT will provide the tools and practices for participants to understand and integrate their psychedelic experiences into their daily life. CFT as a modality is particularly helpful as CFT inherently enhances compassion for self, others, and the world, social connection, and social safeness. Additionally, we also provided an overview of a new treatment protocol for therapists to work with clients within the field of psychedelic psychotherapy.

The integrative approach of psilocybin-assisted CFT circumvents challenges that are associated with the current standard approaches for depression. In real world settings psychotherapies and pharmacotherapies are typically provided by different clinicians, sometimes even at different clinics. However, combining psychotherapy and pharmacotherapies in a collaborative manner is more effective than pharmacotherapy alone (Coventry et al., 2014). The current integrative approach opens the possibility to tailor, adjust, or change the intervention to meet the needs of participants, and thus potentially increase the responsiveness of the participants.

In the current protocol, we believe that CFT is a suitable psychotherapeutic framework for psilocybin treatment of depression. However, other approaches may also offer a relevant psychotherapeutic framework for psilocybin assisted-psychotherapy, such as CBT, ACT, or MET (Bogenschutz et al., 2015; Johnson et al., 2017; Sloshower et al., 2020; Watts and Luoma, 2020). For example, Acceptance and Commitment Therapy (ACT) has been suggested as a framework for major depressive disorders, wherein the focus of the therapy lies on increasing psychological flexibility (Sloshower et al., 2020). CFT as well as ACT focus on changing the relationship of the participants to their thoughts, sensations, and emotions. However, CFT focuses specifically on developing compassion and social connection as a mediator of change and this could be especially suitable for people who have a compromised capacity for experiencing and expressing affiliative motives and emotions, i.e., those with high levels of self-criticism and shame. Future research should examine different mechanisms of change in relation to participants’ physiological characteristics and hopefully can give some guidelines on what treatment protocol best suits whom.

We further believe that particularly CFT is suitable as the psychotherapeutic framework for psilocybin-assisted psychotherapy as it focuses on stimulating motivational systems that evolved to support caring connections. As both CFT and psilocybin-assisted therapy are stimulating connectedness and social safeness, they can augment each other and provide a solid framework for the treatment of depression. Research indicates that some individuals have difficulties with developing compassion, particularly those who have an insecure attachment style and high self-criticism (Kamboj et al., 2015; Kirby, 2017; Steffen et al., 2021). They can experience negative impacts of compassionate imagery, partly because compassion can stimulate the caring system which may have trauma memories. Likewise, in a study by Rockliff et al. (2011) the authors studied the effects of intranasal oxytocin on compassion focused imagery (CFI). They found that participants higher in self-criticism, lower in self-reassurance, social safeness, and attachment security had fewer positive experiences of CFI under oxytocin than placebo, indicating that the effects of oxytocin on affiliation may depend on attachment and self-evaluative styles. It’s possible that psilocybin-assisted psychotherapy can stimulate sadness in that particular group by relating to a deep interconnectedness through stimulating the 5-HT2A receptor pathways (Van De Kar et al., 2001; Holze et al., 2021). Offering an integrated approach of psilocybin-assisted CFT has the potential to break through some of the fears, blocks, and resistances to compassion that are common in therapy, and therefore improve people’s capacity to experience a sense of caring, connectedness, and compassion. Future studies should research the degree in which offering an integrated approach is augmenting the effect of each modality by itself, especially for those who have an insecure attachment style and high self-criticism.

### Limitations

The presented treatment protocol for psilocybin-assisted CFT holds promise, but some limitations also apply. First, it remains unclear if and how much therapeutic guidance is necessary for the effect of psilocybin on depression demonstrated in the recent clinical trials. The underlying assumption is that the navigation sessions (psilocybin administration) open a therapeutic window that disrupts the entrenched negative framework of thoughts, sensations, and emotions, allowing insights or experiential knowledge to arise, that—with psychotherapeutic guidance—can lead to an adjustment into a more balanced emotion regulation system. However, future research should examine the dosage-effect of psychotherapy in psychedelic-assisted psychotherapy (Horton et al., 2021). Now that psychotherapeutic treatment protocols are being published, different protocols could be compared in future studies to conditions were minimal (non-psychotherapeutic) guidance or only psilocybin is offered. Secondly, this protocol was specifically developed as psilocybin-assisted psychotherapy for depression. Generalization of this protocol to other symptoms is not recommended. Also, this treatment protocol should not be used with other psychedelics or medication. Third, we cannot make any claims about the specific competencies needed to administer a psilocybin-assisted protocol for depression. However, we believe, it is crucial for therapists to acquire training in the competencies that are regarded as essential for psychedelic-assisted psychotherapy (Phelps, 2017).

## Conclusion and Future Directions

This paper provides a psychotherapeutic framework for the psilocybin-assisted psychotherapy of depression. As treatment protocols have been scarce so far, the psilocybin-assisted CFT protocol not only fills a gap but also enhances the effectiveness of the psilocybin-assisted therapy. The efficacy of the protocol should be examined, and we have suggested a design that is feasible for interested researchers and/or clinicians.

## Data Availability Statement

The original contributions presented in the study are included in the article/supplementary material, further inquiries can be directed to the corresponding author/s.

## Author Contributions

WP and FC conceived the presented idea. WP developed the theory and treatment protocol. FC designed the study protocol. Both authors contributed equally in writing the final manuscript.

## Conflict of Interest

The authors declare that the research was conducted in the absence of any commercial or financial relationships that could be construed as a potential conflict of interest.

## Publisher’s Note

All claims expressed in this article are solely those of the authors and do not necessarily represent those of their affiliated organizations, or those of the publisher, the editors and the reviewers. Any product that may be evaluated in this article, or claim that may be made by its manufacturer, is not guaranteed or endorsed by the publisher.

## Footnotes

1
psychedelicstoday.com/music-resources/
